# Grants to Extra-Metropolitan Hospitals

**Published:** 1908-12-26

**Authors:** 


### GRANTS TO EXTRA-METROPOLITAN HOSPITALS.

With the sanction of H.R.H. the Prince of Wales, Grand President, the following grants have been made for 1908 to extra-metropolitan hospitals by the League of Mercy:—

Bedfordshire: Bute Hospital, Luton, £60.

Berkshire: Royal Berkshire Hospital, Reading, £200; Windsor and Eton Royal Dispensary, £25; Newbury Children's Cottage Hospital, £5; St. Luke's Home, Woodley, Reading, £5.

Buckinghamshire: Royal Bucks Hospital, Aylesbury, £50; Marlow Cottage Hospital, £5.

Essex: Essex and Colchester General Hospital, £25; Chelmsford Infirmary, £10; Romford, Victoria Cottage Hospital, £10; Braintree and Bocking Cottage Hospital, £5; Brentwood Cottage Hospital, £5; Brentwood Convalescent Home for Children, £5; Buckhurst Hill Village Hospital, £5.

Hampshire: Royal South Hants Hospital, Southampton, £150; Royal Hants County Hospital, Winchester, £150; Royal Portsea, Portsmouth, and Gosport Hospital, £100.

Hertfordshire: Bushey Heath Cottage Hospital, £5.

Kent: West Kent General Hospital, Maidstone, £50; Gravesend Hospital, £30; General Kent and Canterbury Hospital, £25; Tunbridge Wells General Hospital, £25; Folkestone Victoria Cottage Hospital, £15; Ashford Cottage Hospital, £10; Sevenoaks Hospital for Children with Hip Disease, £10; Beckenham Cottage Hospital, £10; Bromley Cottage Hospital, £10; Margate Victoria Home for Children, £10; Faversham Cottage Hospital, £5; Tunbridge Wells Homœopathic Hospital, £5.

Middlesex: Enfield Cottage Hospital, £10; Hounslow Hospital, £10; Hanwell Hospital, £5; Hayes Cottage Hospital, £5; Twickenham St. John's Hospital, £5.

Oxfordshire: Radcliffe Infirmary, Oxford, £150.

Suffolk: Beccles Hospital, £10.

Surrey: Royal Surrey County Hospital, £75; Croydon General Hospital, £25; Cranleigh Village Hospital, £10; Egham Cottage Hospital, £10; Surbiton Cottage Hospital, £10; Woking, Victoria Cottage Hospital, £10; Caterham Cottage Hospital, £5; East and West Molesley Cottage Hospital, £5; Epsom and Ewell Cottage Hospital, £5; Haslemere and District Cottage Hospital, £5; Kingston Victoria Hospital, £5; Leatherhead Victoria Memorial Cottage Hospital £5; Limpsfield Convalescent Home for Women and Children, £5; Purley Cottage Hospital, £5; Sutton Hospital, £5; Thames Ditton Cottage Hospital, £5.

Sussex: Brighton Sussex County Hospital, £50; Hastings, St. Leonards, and East Sussex Hospital, £21; Haywards Heath Cottage Hospital, £10; Eastbourne Princess Alice Memorial Hospital, £10; Crawley Cottage Hospital, £5; Horsham Cottage Hospital, £5.

Captain E. M. Maitland, a member of the *Daily Graphic* Balloon Expedition to Russia, writes that during the time they were in the basket of the balloon (32 hours in all) they were exposed to severe cold—at one time the thermometer registered 4° below zero, the altitude being 17,200 feet, considerably higher than any mountain in Europe; but that their Thermos bottles and cooker enabled them to have hot meals and drinks. The use of these appliances considerably reduced their many hardships, and contributed largely to the success of the expedition.

